# A database of human genes and a gene network involved in response to tick-borne encephalitis virus infection

**DOI:** 10.1186/s12862-017-1107-8

**Published:** 2017-12-28

**Authors:** Elena V. Ignatieva, Alexander V. Igoshin, Nikolay S. Yudin

**Affiliations:** 1 Laboratory of Evolutionary Bioinformatics and Theoretical Genetics, The Federal Research Center Institute of Cytology and Genetics of Siberian Branch of the Russian Academy of Sciences, Novosibirsk, 630090 Russia; 2 Laboratory of Infectious Disease Genomics, The Federal Research Center Institute of Cytology and Genetics of Siberian Branch of the Russian Academy of Sciences, Novosibirsk, 630090 Russia; 3 Center for Brain Neurobiology and Neurogenetics, The Federal Research Center Institute of Cytology and Genetics of Siberian Branch of the Russian Academy of Sciences, Novosibirsk, 630090 Russia; 4 Novosibirsk State University, Novosibirsk, 630090 Russia

**Keywords:** Tick-borne encephalitis, TBEV, *Flavivirus*, Candidate genes, Network, PPIs, Database

## Abstract

**Background:**

Tick-borne encephalitis is caused by the neurotropic, positive-sense RNA virus, tick-borne encephalitis virus (TBEV). TBEV infection can lead to a variety of clinical manifestations ranging from slight fever to severe neurological illness. Very little is known about genetic factors predisposing to severe forms of disease caused by TBEV. The aims of the study were to compile a catalog of human genes involved in response to TBEV infection and to rank genes from the catalog based on the number of neighbors in the network of pairwise interactions involving these genes and TBEV RNA or proteins.

**Results:**

Based on manual review and curation of scientific publications a catalog comprising 140 human genes involved in response to TBEV infection was developed. To provide access to data on all genes, the TBEVhostDB web resource (http://icg.nsc.ru/TBEVHostDB/) was created. We reconstructed a network formed by pairwise interactions between TBEV virion itself, viral RNA and viral proteins and 140 genes/proteins from TBEVHostDB. Genes were ranked according to the number of interactions in the network. Two genes/proteins (*CCR5* and *IFNAR1*) that had maximal number of interactions were revealed. It was found that the subnetworks formed by *CCR5* and *IFNAR1* and their neighbors were a fragments of two key pathways functioning during the course of tick-borne encephalitis: (1) the attenuation of interferon-I signaling pathway by the TBEV NS5 protein that targeted peptidase D; (2) proinflammation and tissue damage pathway triggered by chemokine receptor CCR5 interacting with CD4, CCL3, CCL4, CCL2. Among nine genes associated with severe forms of TBEV infection, three genes/proteins (*CCR5*, *IL10*, *ARID1B)* were found to have protein-protein interactions within the network, and two genes/proteins (*IFNL3* and the *IL10,* that was just mentioned) were up- or down-regulated in response to TBEV infection. Based on this finding, potential mechanisms for participation of *CCR5*, *IL10*, *ARID1B*, and *IFNL3* in the host response to TBEV infection were suggested.

**Conclusions:**

A database comprising 140 human genes involved in response to TBEV infection was compiled and the TBEVHostDB web resource, providing access to all genes was created. This is the first effort of integrating and unifying data on genetic factors that may predispose to severe forms of diseases caused by TBEV. The TBEVHostDB could potentially be used for assessment of risk factors for severe forms of tick-borne encephalitis and for the design of personalized pharmacological strategies for the treatment of TBEV infection.

**Electronic supplementary material:**

The online version of this article (10.1186/s12862-017-1107-8) contains supplementary material, which is available to authorized users.

## Background

Tick-borne encephalitis (TBE) is a severe neurological illness caused by tick-borne encephalitis virus (TBEV). TBEV is a neurotropic, positive-sense RNA virus that belongs to the genus *Flavivirus*, family *Flaviviridae*. TBEV infection can lead to a variety of clinical manifestations ranging from slight fever to severe neurological illness. Infections with TBEV may result in encephalitis, meningitis and haemorrhagic fevers with high mortality rates [1].

TBEV occurs in forest and forest-steppe zones in the territory of central Europe, the Baltic and Scandinavian countries, and the Russian Federation. In Russia, TBE is endemic from Kaliningrad to Vladivostok [2, 3]. Three different subtypes of TBEV have been recognized (European, Siberian, and Far Eastern), which are associated with different disease severity [3].

From 10,000 to 15,000 clinical cases are registered annually in Europe and Asia, [4]. The incidence of TBE in all endemic regions of Europe has increased by almost 400% in the last 30 years. Thus, TBE has become a growing public health challenge in Europe and some other parts of the world [3].

The spectrum of clinical presentations ranges from simple fever to severe encephalitis with or without myelitis. Infection may result in death (0.5–2.0%, case fatality rate possibly higher for the Siberian subtype) or long-term neurological sequelae (up to 58%, according to the World Health Organization) [4]. Although effective vaccines against TBE are available, and are on the market since the 1980s, today there is no specific treatment for infection [5, 6].

Risk factors of the appearance of severe forms of tick-borne encephalitis are poorly understood. Severe forms of the disease can arise both as a result of weakening of antiviral immunity (that leads to an increase in the amount of virions and affection of larger amount of host cells) and due to excessive host immune response [7]. Convincing evidences support the hypothesis that genetic factors may contribute to susceptibility or resistance to flaviviruses [8–11]. Nevertheless, by now, only few studies have been done on the genetic predisposition to severe forms of TBE.

It has been shown in the Russian population that five SNPs in *OAS2* and *OAS3* genes, as well as two SNPs in *IFNL3/IL28B* gene and polymorphisms in the *TLR3, CD209*, and *IL10* genes were associated with predisposition to severe forms of tick-borne encephalitis [12–15]. Polymorphisms in chemokine receptor 5 (*CCR5*) and toll-like receptor 3 (*TLR3*) genes were found to be a risk factors for clinical tick-borne encephalitis in the Lithuanian population [16–18].

Evidence of genetic factors predisposing to diseases caused by TBEV and other closely related flaviviruses (West Nile virus, dengue virus, yellow fever virus, etc.) has been summarized in recent reviews [10, 19–22]. In all cases described above the estimation of the genetic risk of susceptibility to TBE relied on association studies, in which frequencies of candidate gene variants were compared in patients and healthy controls. We did not find any scientific reports based on high-throughput DNA sequencing or high-performance genotyping of samples from TBEV infected patients in available resources.

Like other Flaviviruses, TBEV possesses a positive sense RNA genome that encodes a single polyprotein, which is co- and posttranslationally processed into three structural proteins (Capsid, prM, and Envelope) and seven non-structural proteins (NS1, NS2A, NS2B, NS3, NS4A, NS4B, and NS5) [23–25]. According to the 10th Online Report of the International Committee on Taxonomy of Viruses (ICTV, www.ictvonline.org), the genus *Flavivirus* includes more than 60 virus species of which 40 are known to cause disease in humans [https://talk.ictvonline.org/ictv-reports/ictv_online_report/]. Japanese encephalitis virus, dengue virus, yellow fever virus, West Nile virus, and Zika virus are among the most well-known human pathogens from *Flavivirus* genus [26]. The most closely related viruses from the Tick-borne encephalitis virus serocomplex are Omsk hemorrhagic fever virus, Louping ill virus, and Langat virus [https://talk.ictvonline.org/ictv-reports/ictv_online_report/positive-sense-rna-viruses/w/flaviviridae].

At present diverse screening techniques have been applied providing unbiased data on host-pathogen interactions during viral infection. The growing number of studies provided a wealth of information regarding viruses from the *Flaviviridae* family, including screening of the host response to such closely related viral species as West Nile virus [27], and Langat virus [28]. Genome-wide association study aimed at the identification of the susceptibility loci for dengue shock syndrome (caused by the dengue virus) was performed [29].

Despite the enormous value of data obtained for related viral species (West Nile, dengue, Langat viruses) there is a gap in the knowledge about critical contact points between TBEV and host cells (from the human or closely related mammalian organisms). Evidences exist that each viral species interferes with the unique repertoire of host factors to promote infection [30–32]. Additionally, each viral species has developed its own mechanisms to avoid the host immune response [33–35]. Thus, any piece of evidence of involvement of a gene or protein in response to TBEV infection may be valuable. Compiling data on genes involved in response to TBEV infection and integrating them in online resource may facilitate identification of potential drug targets and development of novel strategies for treating infection caused by TBEV.

The objectives of this study were: (1) to compile a catalog of human genes involved in response to TBEV infection; (2) to construct and analyze the network formed by pairwise physical interactions between genes/proteins from the catalog and their pairwise interactions with TBEV; (3) to rank genes according to the number of neighbors in the network.

## Methods

### Compiling candidate genes and assigning them to functional categories

Firstly, candidate genes were selected using a domain-specific search engine for medical information Coremine Medical (www.coremine.com), which offers networks involving genes and proteins related to query term(s). *Tick-Borne Encephalitis’ (disease)* was used as a search term. For each gene identified from the Coremine Medical tool, we performed manual literature mining to find research articles confirming involvement of genes in response to TBEV infection.

We revealed that a number of publications reviewed at the first step presented evidences confirming involvement of additional other genes (not found by Coremine Medical) in response to TBEV infection.

For this reason, online searches were then undertaken (PubMed), using the following combinations of the key words: (1) (*TBEV* OR *tick-borne encephalitis*) AND (*PPI* OR *Physical interactions*); (2) (*TBEV* OR *tick-borne encephalitis*) AND *expression*; (3) (*TBEV* OR *tick-borne encephalitis*) AND *association*; (4) (*TBEV* OR *tick-borne encephalitis*) AND *Knockout*. This yielded a collection of research articles describing involvement of human genes/proteins (or genes/proteins from other mammalian species) in response to TBEV. In accordance with the type of evidence found in the article each gene/protein was assigned to a specific category (dataset). The names of datasets and their descriptions are presented in the Table 1.

**Table 1:** Functional groups of genes/proteins (datasets) that were included into the catalog of genes involved in response to TBEV infection

	Dataset (type of evidence)	Description of the dataset	Number of genes	Number of publications
1.	Physical interaction (Additional file 1: Table S1)	Genes encoding proteins that had direct physical interactions with TBE viral particle, TBEV proteins or RNA.	51	13
2.	Up- or down-regulated (Additional file 1: Table S2)	Genes encoding mRNAs (or proteins) that were up- or down-regulated in response to TBEV infection^a^	76	36
3.	Allelic variant (Additional file 1: Table S3)	Allelic variant in this gene was associated with susceptibility or resistance to TBEV infection^b^	9	6
4.	Increased/attenuated antiviral activity (Additional file 1: Table S4)	These proteins were required for inhibitory effect of other proteins against TBEV or attenuated its antiviral activity	6	6
5.	Knockout (Additional file 1: Table S5)	Knockout of these genes in mice increased mortality rates or affected the other clinical manifestations of the disease	6	4
	All_catalog (Additional file 1: Table S6)	The catalog of human genes involved in response to TBEV infection.	140	53

^a^If there was evidence that the level of an active form of the protein changed in response to TBEV infection, the gene encoding such protein was also included into this dataset

^b^That meant that clinical severity of disease or some immunological parameters in patients with TBE were associated with one of allelic variants

To create a catalog of genes involved in response to TBEV infection we merged all datasets and removed duplicates.

### Network construction

Using data extracted from the literature, the following six types of pairwise interaction were generated, involving genes or proteins from the catalog: (1) physical interactions between viral proteins or RNA or the whole TBEV particle and host proteins (*PIs involving TBEV*); (2) the effects of TBE viral particle or viral RNA or TBEV proteins on the expression levels of the host mRNA or proteins (*Up- or down-regulation*); (3) associations of allelic variants in human genes with susceptibility or resistance to TBEV infection (*Associations)*; (4) physical interactions with proteins from the first three groups named above affecting the biological response to TBEV (*PPIs affecting response)*; (5) indirect interaction with proteins from the first three groups named above within the same signaling pathway affecting the biological response to TBEV (*Interaction within pathway)*; (6) the effect of a gene knockout on the survival time after TBEV infection (*Knockout).* The description of pairwise interactions is presented in Table 2.

**Table 2:** Pairwise interactions in the network involving genes/proteins from the catalog

	Short name of the interaction	Description	Node 1	Node 2	Data source	Number of interactions in the network
1.	PIs involving TBEV	Physical interactions between viral proteins or RNA or the whole TBEV particle and host proteins	The whole TBEV particle, or viral proteins or viral RNA	Human gene/protein from the catalog	Research articles	51
2.	Up- or down-regulation	The effects of the whole TBEV particle or viral RNA or TBEV proteins on the expression levels of the host mRNA or proteins	The whole TBEV particle, or viral proteins or viral RNA	Human gene/protein from the catalog	Research articles	76
3.	Associations	Associations of allelic variants in human genes with susceptibility or resistance to TBEV infection	The object TBEV susceptibility/resistance	Human gene/protein from the catalog	Research articles	9
4.	PPIs affecting response	Physical interactions with proteins from the catalog affecting the biological response to TBEV	Human gene/protein from the catalog	Human gene/protein from the catalog	Research articles	4
5.	Interaction within pathway	Indirect interaction with proteins from the catalog within the same signaling pathway affecting the biological response to TBEV	Human gene/protein from the catalog	Human gene/protein from the catalog	Research articles	2
6.	Knockout	The effect of a gene knockout on the survival time after TBEV infection or disease manifestations	The object Knockout	Human gene/protein from the catalog	Research articles	6
7.	PPI_STRING (Additional file 1: Table S7)	Physical interactions between genes/proteins from the catalog obtained from STRING and passed manual verification	Human gene/protein from the catalog	Human gene/protein from the catalog	STRING and Research articles	25

We also employed the STRING (Search Tool for the Retrieval of Interacting Genes/Proteins) [36] to identify pairwise physical interactions between all human proteins encoded by genes compiled in the catalog. We considered physical interactions with STRING scores greater than 0.4. Beyond that all pairwise physical interactions obtained from STRING were checked manually and only those interactions were selected which were described in research articles and were revealed in human or rodents. Thus, we obtained data on the seventh type of pairwise interactions *PPI_STRING* (Table 2). Data on interactions *PPI_STRING* are presented in the Additional file 1: Table S7.

To construct a network integrating genes/proteins involved in response to TBEV infection, data on pairwise relationships of all types described above (Table 2) were imported into Cytoscape [37].

The following data had been imported into Cytoscape as attributes of nodes and edges: (1) the functional category of each gene/protein (the datasets, described in the Table 1); (2) the type of pairwise interactions between objects in the network (*physical interactions, up- or down-regulation, Associations*, etc., described in the Table 2). These attributes of nodes and edges were used to arrange the visualization style.

## Results

### The catalog of human genes involved in response to tick-borne encephalitis virus infection

By systematic review and curation of multiple lines of evidence we created a catalog of human genes involved in response to TBEV infection. Among them 44 genes were initially obtained from Coremine Medical tool and the other 96 genes were found in scientific publications manually. As a result, we selected 140 candidate genes (Table 1, and Additional file 1: Table S6). The number of genes classified into functional categories (datasets) according to five types of evidence listed in the Compiling candidate genes and assigning them to functional categories section are presented in the Table 1.

*The physical interaction* dataset included 51 genes (Additional file 1: Table S1). Three proteins in the *Physical interaction* dataset (laminin subunit beta 1, extracellular matrix protein encoded by *LAMB1,* integrin subunit alpha 3 encoded by *ITGA3*, and ribosomal protein SA (also known as 67 kD laminin receptor) encoded by *RPSA*) were revealed to have interactions with the whole TBE viral particle [38]. Two proteins (TIA1, cytotoxic granule associated RNA binding protein (encoded by *TIA1*), and TIAL1 cytotoxic granule associated RNA binding protein like 1 (encoded by *TIAL1*)) were found to interact with the viral RNA [39]. In addition, one protein (immunoglobulin-like cell surface protein ILT7 encoded by *LILRA4*) interacted with inactivated whole virus vaccine against TBE (FSME-IMMUN) [40]. The largest portion of proteins (47 out of 51) was found to interact with the individual viral proteins (prM, NS5, and E). Moreover, 44 out of these 47 proteins interacted with the viral protein NS5. Most proteins interacting physically with TBEV NS5 protein (33 out of 47), were compiled from one research article based on data obtained from a high-throughput yeast two-hybrid screen [30].

*Up- or down-regulated* dataset included 76 genes (Additional file 1: Table S2). For 40 genes from *Up- or down-regulated* dataset we found expression data at the level of mRNA, and for 49 genes we found that the level of encoded proteins or its active forms increased or decreased in response to TBEV infection. Besides, 39 out of 76 genes/proteins from *Up- or down-regulated* dataset were revealed from in vivo studies comparing TBEV-infected and uninfected human sera or other human biological samples (plasma, cerebrospinal fluid (CSF), etc.) (Table 3). The levels of eight, ten and two proteins were found to be up- or down-regulated in the sera, or plasma, or blood of infected patients. The levels of ten and two proteins were changed in CSF or liquor of infected patients. In addition, ten and 13 proteins changed their levels in NK cells and T cells of TBEV-infected patients.

**Table 3:** Genes/proteins from *Up- or down-regulated* dataset that were revealed from studies comparing TBEV-infected and uninfected human sera or other biological samples

Biological sample	Genes	References
Serum	ICAM2	[87]
	MMP9	[88]
	ICAM3, ICAM1	[89]
	IL10, IFNB1	[46]
	CXCL10, CXCL13	[90]
Plasma	IFNG, TNF, IL6, CXCL8, IL2, IL12A, IL12B, IL15, IL18, IFNA1	[91]
Blood	LTF	[92]
	GSN	[93]
CSF	IFNL3, IL10, IFNB1	[46]
	ICAM1	[87, 89, 94]
	ICAM2	[89]
	ICAM3	[87, 89]
	CXCL10, CXCL11, CXCL12, CXCL13	[90]
Liquor	A2M	[67]
	LTF	[92]
NK cells	MKI67, BCL2, PRF1, GZMB, IL2, IL12A, IL12B, IL15, IL18, IFNA1	[91]
T cells	PRF1, PDCD1, BCL2, GZMB, IL7R, CD27, TBX21, EOMES, IKZF2	[95]
	IL5	[96]
	IFNG	[96, 97]
	IL2, TBX21	[97]

*Allelic variant* dataset included nine genes (Additional file 1: Table S3). The clinical severity of disease or some immunological parameters in patients with TBE were associated with one of allelic variants in these genes. Data was collected on 12 SNP and one 32-base-pair deletion (CCR5delta32) located not only in the bodies of these genes but also in their 5′- or 3′-flanking regions, as it is well known that upstream and downstream gene regions are very important for transcriptional regulation [41–45].

Nine out of 13 SNPs (in *TLR3, CD209, OAS2, OAS3, IFNL3/IL28B,* and *IL10*) were studied in Russian population from Novosibirsk [12–15]. One SNP (rs3775291 in the 4th exon of *TLR3*) and one 32-base-pair deletion (rs333) in *CCR5* coding region (CCR5delta32) were studied in Lithuanian population [18]. In addition, two SNPs (rs12979860 in the first intron of *IFNL4* and rs287886 the first intron of *ARID1B*) were studied in the Polish population [46].

It should be noted that according to [46], rs12979860 locus (located in the first intron of *IFNL4* and upstream of IFNL3) was associated with *IFNL3* expression. The second polymorphic locus rs287886 described in this report was found to be associated with *IL10* expression [46]. These authors annotated the second polymorphic locus (rs287886) to *CD209* gene located on 19 chromosome. However, according to dbSNP, rs287886 is located in the first intron of *ARID1B* gene mapped to the 6-th chromosome. Thus, we included *IFNL3* and *IL10* into the *Up- or down-regulated* dataset. *IFNL4* and *ARID1B* were included into *Allelic variant* dataset. *CD209* gene was also included into the *Allelic variant* dataset based on data reported in [13]. In this study it was revealed that the rs2287886 SNP in the *CD209* promoter region (but not rs287886 that was annotated to *CD209* gene by [46]), was associated with predisposition to severe forms of TBE in the Russian population.

*Increases/attenuates antiviral activity* dataset included six genes/proteins (Additional file 1: Table S4). Four out of six proteins (encoded by *RAC1, ARHGEF7, CIAO1, ERN1*) exerted activation or attenuation via direct physical interactions with the other proteins [47–49]. The other two proteins (encoded by *S1PR4* and *PEPD*) collaborated with other proteins with well-known antiviral activity indirectly, being involved in the same signaling pathway [40, 50].

The *Knockout* dataset included six genes/proteins (Additional file 1: Table S5). Knockout of these genes in mice (1) increased mortality rates (*MAVS, TNF*) [51, 52]; (2) delayed the appearance of neurological signs of disease (*CD8A*) [53]; (3) affected (increased or decreased) TBEV extracellular infectivity (*TIA1, TIAL1*) [39].

To determine the total number of genes involved in response to TBEV infection, we merged all gene sets. With duplicates removed, a list comprising 140 unique genes was obtained (Table 1 and Additional file 1: Table S6).

### The TBEVHostDB web resource

To provide access to genes from the catalog, the TBEVhostDB web resource (http://icg.nsc.ru/TBEVHostDB/) was created. In addition to the full list of genes, TBEVhostDB includes five lists of functional groups of genes that were described above (Sections Compiling candidate genes and assigning them to functional categories and The catalog of human genes involved in response to tick-borne encephalitis virus infection, and Table 1). References to scientific publications and active links to NCBI resources (EntrezGene, PubMed, dbSNP) are given. In addition, some experimental details (host organism, method (in vivo/in vitro), TBEV strain (if available), etc.) are also presented in TBEVhostDB.

### Network formed by pairwise interactions between genes/proteins

**Fig. 1:**
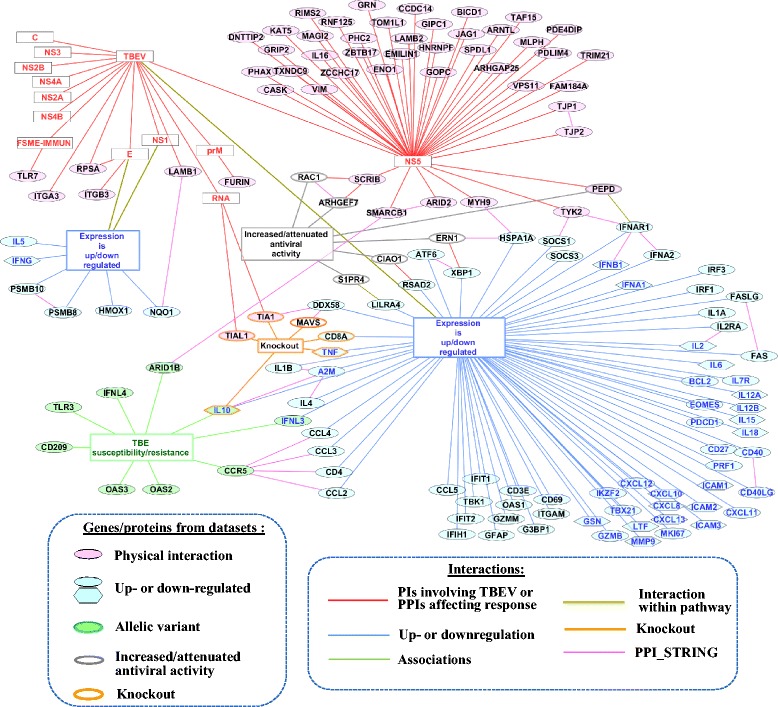
The network presenting data on pairwise interactions involving TBEV (or viral RNA and protein) and 140 human genes/proteins from the TBEVHostDB. The descriptions of genes/proteins datasets were given in Table 1. The descriptions of interactions were given in Table 2. The names of 39 genes/proteins from *up- or down-regulated* dataset that were revealed from studies comparing TBEV-infected and uninfected human sera or other human biological samples (presented in Table 3) are shown in blue. Hexagons denote 20 proteins revealed to be up- or down-regulated in human sera, plasma, and blood

To visualize pairwise interactions between genes/proteins from the TBEVHostDB, a network presenting data on interactions extracted from the literature and the STRING database was reconstructed. We classified pairwise interactions extracted from scientific publications into six categories (types) that were described previously in the section Network construction. The seventh category included direct physical interactions between human proteins that were obtained from STRING and that passed a manual verification. According to STRING, 39 proteins/genes from the catalog were involved in pairwise physical interactions with each other (Additional file 1: Table S7). The numbers of interactions of each type are presented in the Table 2. All data was uploaded into Cytoscape [37]. Thus, a network comprising pairwise interactions involving TBEV (or viral RNA and protein) and 140 human genes/proteins from the TBEVHostDB was constructed (Fig. 1). About 30 % of genes from the network (39 out of 140, in the Fig. 1 these gene names are shown in blue) were revealed from studies comparing TBEV-infected and uninfected human sera or other human biological samples (presented in Table 3). The levels of 20 proteins (denoted by hexagons) were found to be changed in TBEV-infected human sera (or plasma and blood of TBEV-infected patients).

The data presented in a visualization-ready format that allows the direct re-creation of the interactive version of the Fig. 1 with Cytoscape is presented in the Additional file 2.

### The ranking of genes in the network of pairwise interactions involving TBEV and 140 human genes/proteins from the TBEVHostDB

**Fig. 2:**
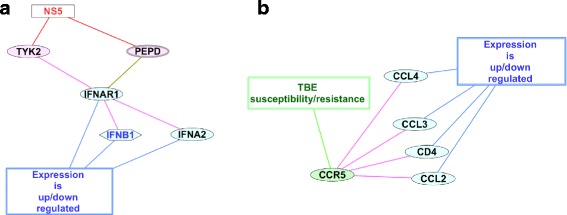
The subnetworks formed by human proteins with the maximal number of neighbors. Panel (A) IFNAR1 and its neighbors; Panel (B) CCR5 and its neighbors. The color legend is as described in Fig. 1

We ranked human genes/proteins in the network according to the number of pairwise interactions with the other human genes/proteins from the TBEVHostDB. It was found that 41 human proteins (~29%) had one or more pairwise interactions with the other human proteins (Additional file 1: Table S8). Among them, 29 proteins had one interaction with the other human proteins. Ten proteins had two interactions. And only two proteins (IFNAR1 and CCR5) had four interactions (with TYK2, PEPD, IFNB1, IFNA2 and CD4, CCL3, CCL4, CCL2, respectively) (Table 4). The subnetworks formed by IFNAR1 and CCR5 and their first neighbors are presented in Fig. 2a and b.

**Table 4:** Genes/proteins from the TBEVHostDB that had four pairwise interactions with the other genes/proteins within the network

The central gene/protein	Dataset/Type of evidence	Number of neighbors	Neighbors	Biological role of subnetwork
IFNAR1	Up- or down-regulated	4	TYK2, PEPD, IFNB1, IFNA2	Attenuation of interferon signaling pathway by viral protein NS5 targeting PEPD
CCR5	Allelic variant	4	CD4, CCL3, CCL4, CCL2	The entry of TBEV into the host cell/The proinflammatory and tissue damage pathway

## Discussion

### A catalog of human genes involved in response to TBEV infection and the TBEVHostDB web resource

The main objective of the present study was to obtain a systematic overview of human genes involved in response to TBEV infection. These genes may serve as clinical biomarkers for prediction of the TBEV infection course and outcome in humans. A systematic review of the literature revealed genes that were relevant to response to TBEV infection. As a result a catalog comprising 140 human genes was created (Table 1, and Additional file 1: Tables S1-S6), and the TBEVHostDB web resource was designed. Thirty percent of genes from the TBEVHostDB (39 genes) were revealed from studies comparing TBEV-infected and uninfected human sera or other human biological samples (Table 3). We did not find any analogs for such a comprehensive catalog of human genes relevant to response to TBEV infection. To date, genome-wide association studies have never been applied for identification of the genes associated with human genetic predisposition to TBE [54]. The most recent review on genetic predisposition to diseases caused by flaviviruses presents nine human genes possessing allelic variants associated with severe forms of TBEV infection [22]. The host cell response to tick-borne encephalitis virus was described recently [55].

The other most comprehensive scientific report in this field described the knowledge base VirHostNet (http://pbildb1.univ-lyon1.fr/virhostnet) for the management and the analysis of proteome-wide virus-host interaction networks. To date, this knowledge base included data on 35 human genes encoding proteins involved in direct physical interactions with TBEV proteins [56]. Thus, the TBEVHostDB web resource, created by manual curation of scientific publications, is the first effort of integrating and unifying data on genes/proteins involved in response to TBEV infection and genetic factors that may predispose to severe forms of diseases caused by TBEV.

### Networks formed by associations between genes/proteins and ranking genes

At the next step, a network presenting pairwise interactions between TBEV particle itself, viral RNA and viral proteins and 140 genes/proteins from the TBEVHostDB was reconstructed (Fig. 1, Additional file 2). The network was formed by 173 interactions of seven types (Table 2). Interactions of the first six types (*PIs involving TBEV, Up- or down-regulation, Associations, PPIs affecting response, Interaction within pathway, Knockout*) were obtained manually from the scientific publications. The data on physical interactions *PPI_STRING* involving human genes/proteins from the catalog was extracted from the STRING database [36]. Thus, it allowed us to add additional 25 pairwise interactions involving 39 human genes/proteins within the network (Additional file 1: Table S7).

We ranked genes/proteins in the network according to the number of pairwise interactions and revealed two proteins (IFNAR1, CCR5) with the maximal number of interactions (each protein had four interactions) (Table 4, and Additional file 1: Table S8). Thus, two subnetworks formed by these proteins and their closest neighbors (Fig. 2a and b) were identified. A hypothesis on the functioning of subnetworks in the context of the host response to TBEV infection is as follows:

#### IFNAR1 subnetwork

IFNAR1 (interferon alpha and beta receptor subunit 1) had three direct physical interactions extracted from STRING (with tyrosine kinase 2 (*TYK2),* interferon beta 1 *(IFNB1),* interferon alpha 2 *(IFNA2*)) and one indirect interaction (*interaction within pathway*, extracted from the research article [50]) with peptidase D (PEPD) (Fig. 2a). It is well known that all these five proteins (IFNAR1, TYK2*,* IFNB1, IFNA2, and PEPD) are involved in signal transduction pathway activating antiviral response (Interferon signaling cascade, Jak/STAT pathway) [34, 50, 57]. In addition, two proteins TYK2 and PEPD had direct interactions with the viral NS5 protein [30, 50]. The expression levels of the other two genes/proteins (IFNB1, IFNA2) as well as IFNAR1 were changed in response to TBEV infection in humans and mice [46, 50, 58]. Thus, we suggest that the subnetwork formed by NS5, from one hand, and IFNAR1, TYK2, IFNB1, IFNA2 and PEPD, from the other hand, represents the specific mechanism utilized by TBEV for interfering antiviral response of the host cell.

According to [50] TBEV antagonism of the type I interferon signaling revealed PEPD as a regulator of IFNAR1 surface expression. NS5 derived from TBEV interacted with PEPD. In turn, PEPD is required for IFNAR1 maturation. This researcher proposed that PEPD might function in IFNAR1 biosynthesis by facilitating its trafficking through the ER-to-Golgi network [50]. Thus NS5 binding to PEPD attenuates its activity, reducing IFNAR1 maturation and its expression on the cell surface.

On the other hand, a direct physical interaction between TBEV protein NS5 and the host tyrosine kinase 2 (TYK2) was identified [30]. TYK2 associates with the cytoplasmic domain of type I and type II cytokine receptors and transmits cytokine signals by phosphorylating receptor subunits [59]. We propose that protein-protein interactions between NS5 and TYK2 may serve as an additional mechanism leading to attenuation of interferon signaling.

#### CCR5 subnetwork

CCR5 had four direct physical interactions with CD4, CCL2, CCL3, and CCL4 (Fig. 2b). All four interactions were extracted from STRING and their reliability was checked manually by reviewing the literature. CCR5 encodes a cell surface receptor from the beta chemokine receptor family and is known to be an important co-receptor for a number of macrophage-tropic viruses including human immunodeficiency virus and simian immunodeficiency virus [60].

On the other hand, CCL2/MCP1, CCL3/MIP-1-alpha, and CCL4/MIP-1-beta are C-C motif chemokine ligands, proinflammatory mediators interacting with C-C chemokine receptors (like CCR1, CCR2, CCR4), and CCR5 is among them [60]. CD4 is a membrane glycoprotein, mediator of indirect neuronal damage in infectious and immune-mediated diseases of the central nervous system, which is able to form a complex with CCR5 in blood monocyte-derived dendritic cells [61]. All four genes/proteins that were found to be involved in pairwise interactions with CCR5, were from *Up- or down-regulated* dataset. In particular, the expression of CCL4/MIP-1beta in primary human brain cortex astrocytes was upregulated in response to TBEV infection [62]. Furthermore the levels of CD4, CCL2/MCP-1, CCL3/MIP-1alpha, CCL4/MIP-1beta mRNAs were increased in brains of BALB/c mice infected with TBEV [63]. An excess release of proinflammatory mediators by the brain in response to TBEV infection may be the cause of tissue damage observed in encephalitis [64].

The examination of the subnetwork formed by CD4, CCL2, CCL3, CCL4, and CCR5 leads to the hypothesis that the CCR5 allelic variant CCR5delta32 (rs333) may affect the TBE outcome not only affecting the entry of TBEV into the cell, but also modulating chemokine activity towards neural cells and CD4 glycoprotein functioning. In favor of this assumption are results obtained in mouse models: (1) in mice infected with West Nile virus, chemokine receptor CCR5 may promote leukocyte trafficking to the brain and host survival [65]; (2) CCR5 enhances lymphocyte activation in mice infected with Japanese encephalitis virus, thereby promoting their survival [66].

### Pairwise interactions involving proteins from allelic variant dataset

Using the STRING database and subsequent manual curation of evidences confirming protein-protein interactions, we revealed 25 physical interactions involving 39 genes/proteins from the TBEVHostDB (Fig. 1, Additional file 1: Table S7). Four of these 39 genes/proteins were from the *Allelic variant* dataset. CCR5 which was revealed as a gene/protein with the maximal number of neighbors in the network was one of these four genes/proteins. In the previous section we proposed that Allelic variant CCR5delta32 in *CCR5* may modulate chemokine activity towards neural cells and CD4 glycoprotein functioning.

The other three genes/proteins from the *Allelic variant* dataset, which were involved in pairwise interactions, were *IL10*, *ARID1B*, and *IFNL3* (Fig. 1).

*IL10* encodes cytokine that was upregulated in brains of mice infected with TBEV [63], as well as in the cerebrospinal fluid and in the serum of patients with TBE [46]. The *IL10* allelic variant in promoter region (rs1800872) was associated with predisposition to TBE in Russian population [15]. The protective role of IL10 against TBEV infection has been demonstrated in KO mice: knockout of *IL10* significantly increased mortality rates in mice infected with TBEV [52]. Using STRING we found that IL10 interacted physically with alpha-2-macroglobulin (A2M) that was also found to be elevated in patients with the meningeal and focal forms of tick-borne encephalitis [67]. Thus, the nucleotide substitution (rs1800872 locus) in the promoter of *IL10* may decrease expression level of interleukin 10, affecting its protective activity against pathological processes caused by TBEV.

*ARID1B* encodes AT-rich DNA interacting domain-containing protein functioning as a component of the SWI/SNF chromatin remodeling complex. From the STRING database we found that human ARID1B (UniProt ID = Q8NFD5) interacted physically with human SMARCB1/SNF5 (UniProt ID = Q12824) [68]. In turn, SMARCB1/SNF5 interacted physically with ARID2/BAF200, and both proteins were found to be components of PBAF chromatin remodeling complex in Hela cells [69]. Moreover, ARID2/BAF200 was required for selective expression of interferon-alpha-inducible genes [69]. Basing on these observations we suggest that nucleotide substitution A- > G (rs287886) in the first intron of *ARID1B* may mark a haplotype that included some exonic nonsynonymous nucleotide substitutions. In turn, substitutions of amino acids in ARID1B may disrupt or attenuate ability of this protein to interact with other components of the PBAF chromatin remodeling complex that may be crucial for interferon response.

*IFNL3/IL28B* is the fourth gene that had allelic variants associated with predisposition to TBE, and had an additional connection in the network (Fig. 1). *IFNL3/IL28B* was also contained in the *Up- or down-regulated* dataset. The associations of allelic variants in two polymorphic loci (rs8103142 and rs12980275) within *IFNL3/IL28B* with predisposition to TBE were revealed in the Russian population from Novosibirsk [15]. Additionally, it was found in the Polish population that the level of IFNL3/IL28B in the cerebrospinal fluid of patients with TBE was significantly higher than in the control group [46]. Based on this finding Grygorczuk S et al. assumed that IFNL3/IL28B might play a protective role in TBE. Thus, we suggest that rs12980275 locus in the IFNL3 3′-flanking region may impair transcriptional regulatory activity of this region that may lead to decreased *IFNL3/IL28B* expression. Ultimately, reduced levels of IFNL3/IL28B may influence the disease outcome.

### The differences in severity of TBE may be caused by the different TBEV subtypes

The TBEVHostDB had been created as a catalog of human genes involved in response to TBEV infection. Thus, TBEVHostDB may be regarded as a database on genetic factors in humans that may potentially play a role in the severity of the disease. Besides this, an increasing number of studies show that the severity of the disease may be determined not only by genetic factors in humans, but also by genetic factors related to the virus subtypes.

As it was mentioned previously, three genetically distinct subtypes of viruses within a single TBE virus serocomplex cause TBE. These three subtypes consist of Far-Eastern subtype, Siberian subtype and European subtype. Each of these subtypes cause clinically distinct diseases with varying degrees of severity [70]. TBEV of European subtype generally causes a biphasic disease, occasionally resulting in neurologic disease, but with a low case fatality rate. In contrast, infection by Far-Eastern subtype of TBEV is more frequently associated with severe neurologic disease, relatively high case fatality rate and an increased propensity for neurological sequelae in survivors. The Siberian subtype of TBEV is intermediate in disease severity, but has been associated with chronic infection [4, 70]. The experiments on animals confirm the opinion that different TBEV subtypes possess different pathogenic activities. First, colonized bank voles were infected by TBEV and the infection kinetics of all three known TBEV subtypes were studied. Throughout all time points post infection, RNA of the Far-Eastern TBEV was detected significantly more often than RNA of the other two strains in all organs studied [71]. Second, the Siberian subtype of the TBEV was different from the Far-Eastern subtype by a moderate virulence observed in Siberian hamsters and by a low infection development rate [72].

The relationship between the structure of the TBEV strains and their virulence or pathogenic properties had been studied for all three TBEV subtypes.

#### European subtype

In the study analyzing 72 TBE viruses of European subtype (sampled in Switzerland) the complete envelope (E) protein sequences were identified and phylogenetic classification was made out. Although all isolates were highly related (mean pairwise sequence identity of 97.8% at the nucleotide and 99.6% at the amino acid level of the E protein), more than half (57.8%) of isolates, that were characterized in vitro with respect to their plaque phenotype, produced a mixture of plaques of different sizes, reflecting a heterogeneous population of virus variants [73].

In a mouse model the role of the poly(A) tract in the replication and virulence of TBEV strain of European subtype Torö-2003 was detected. The TBEV strain Torö-38A (containing modified (A)_3_C(A)_38_ sequence) replicated poorly compared to Torö-6A (containing the wild-type (A)_3_C(A)_6_ sequence) in cell culture, but Torö-38A was more virulent than Torö-6A in a mouse model of TBE [74].

#### Far-Eastern subtype

Recently, complete genomes of 34 Far-Eastern subtype TBEV strains isolated from patients with different disease severity (Primorye, the Russian Far East) were sequenced and compared. It was found that the most pathogenic strains (causing encephalitic form of the disease) were divided into two branches: (1) including those related to the Sofjin strain (isolated in Russia, Primorye); (2) including Senzhang strain (isolated in northern China). Strains from patients with the subclinical form of TBE formed a third separate cluster, including the Oshima strain [75].

The other two studies analyzing pathogenicity of the Far-Eastern subtype of TBEV (Sofjin-HO (highly pathogenic) and Oshima 5–10 (low pathogenic)) revealed the variable region of the 3’ UTR as a critical virulence factor in a mouse model [76, 77].

Different pathogenic potentials of strains belonging to different clusters of phylogenetic tree based on complete genome sequencing of the Far-Eastern TBEV strains was revealed using a model of inbred mice of different ages [78].

Pathologic potential of variant clones of the Oshima strain of Far-Eastern subtype of TBEV was analyzed in a separate study. It was shown that an amino acid substitution of Glu122 → Gly in the E protein could have affected virus infection and replication in vivo, as well as the attenuated pathogenicity in mice [79].

Molecular mechanisms of interaction between human immune cells and Far Eastern TBEV strains (Dal’negorsk strain and Primorye-183 strain) were studied in vitro. The highly pathogenic Dal’negorsk strain penetrated into the blood cells more rapidly than Primorye-183 strain. Moreover, these two strains induced a significantly different changes of adhesion and activation receptors expression levels in monocytes and NK cells [80].

#### Siberian subtype

The experimental infection caused in mice by two variants of one TBEV strain of Siberian subtype (strain EK-328 and variant 58, received from this strain population by cloning one plaque) was studied. The viruses differed from each other by three amino acids in the non-structural region (proteins NS2A and NS4A). It was found that these two strains differed in their effect on lymphocyte subpopulation structure of infected mice, providing different effects [81].

Without claiming to be complete, this section indicates the need for accounting genetic factors related to the virus subtypes in predicting the severity of disease caused by TBEV infection.

## Conclusion

It is known that susceptibility to infectious (and in particular, viral) diseases is a multifactorial trait, controlled by multiple genetic factors in combination with external environmental factors [10, 82–85]. The identification of genes responsible for susceptibility/resistance of the host organism to TBEV infection is an ongoing challenge for modern molecular and medical genetics.

Due to the limited geographical distribution of ticks that carry TBEV (Central Europe, Baltic and Scandinavian countries, and the Russian Federation), the studies devoted to this problem are not numerous (in comparison to the number of studies focused on infections caused by the other viruses from the family *Flaviviridae* (Langat virus, Dengue virus, Japanese encephalitis virus, and, especially, hepatitis C virus)). However, given the fact that TBEV can cause severe infection in humans with a variety of neurological symptoms and diseases, the development of effective approaches to treatment of patients with TBE is crucial.

The evidence suggests that each virus species can interact with a unique set of proteins in the host cells [30, 86]. In accordance with this phenomenon, each virus species has developed its own countermeasures against immune response [33–35].

Based on these observations, it can be concluded that the data on the genes involved in response to other flaviviruses (even closely related to TBEV) do not fully relate to the mechanisms of TBEV interaction with host cell. Therefore, we focused on the task of collecting human genes revealed only in the context of response to TBEV.

As a result, the TBEVHostDB informational resource, comprising 140 human genes involved in response to TBEV infection was created. The reconstruction and analysis of the network formed by pairwise interactions involving genes/proteins from the TBEVHostDB and TBE viral particle (or viral RNA, or viral proteins) enabled us (1) to rank genes according to the number of neighbors, and (2) to reveal two subnetworks with clear biological roles in the context of the response to TBEV infection. Based on research evidence found in the literature [26, 50] we inferred that the first subnetwork formed by IFNAR1 (central node) and TYK2, PEPD, IFNB1, and IFNA2 presents the attenuation of interferon response by TBEV. In addition, we suggested that the second subnetwork (CCR5 as the central node, and its neighbors - CD4, CCL3, CCL4, and CCL2) may be the fragment of the proinflammatory signaling pathway. In addition, potential mechanisms for participation of *CCR5*, *IL10*, *ARID1B*, and *IFNL3* (genes from *Allelic variant* dataset) in the host response to TBEV infection were suggested.

This study aimed to collate all of the previously-published work in this area. Identification and systemization of data on genes involved in the host response to TBEV infection is important for understanding the molecular mechanisms of the interaction of TBE virus with the human body, as well as for identifying individuals at high risk for subsequent individualization of preventive measures and medical treatment. Beyond that, despite the fact that currently there is a human TBEV vaccine available, actually there is no specific treatment once infected. Hence, compiling genes and proteins involved in response to TBEV infection may provide grounds for the development of new therapeutics, which is one of the major concerns of TBEV research.

## Additional files

Additional file 1: Table S1. Human genes/proteins (51) that had direct physical interactions with TBE viral particle, TBEV proteins or RNA. **Table S2.** Human genes (76) encoding mRNAs (or proteins) that were up- or down-regulated in response to TBEV infection. **Table S3.** Human genes (9) that possessed allelic variants associated with susceptibility or resistance to TBEV infection. **Table S4.** Human genes (6) encoding proteins that were required for inhibitory effect of other proteins against TBEV or attenuated its antiviral activity. **Table S5.** Human genes (6): knockout of these genes in mice increased mortality rates or effected the other clinical manifestations of the disease. **Table S6.** All genes from the catalog (140 genes). **Table S7.** Direct physical interactions (25) that were obtained from STRING and passed manual verification. **Table S8.** Genes/proteins from the catalog (41) that had one or more pairwise interactions with human genes/proteins within the network. (XLSX 151 kb)

Additional file 2: Supplementary data for creation of the interactive version of gene network in Cytoscape. (XGMML 224 kb)

## Abbreviations

PIs: Pairwise interactions
PPIs: Protein-protein interaction
SNPs: Single nucleotide polymorphisms
TBE: Tick-borne encephalitis
TBEV: Tick-borne encephalitis virus

## References

[1] Kaiser R (2012). Tick-borne encephalitis: clinical findings and prognosis in adults. Wien Med Wochenschr.

[2] Süss J (2011). Tick-borne encephalitis 2010: epidemiology, risk areas, and virus strains in Europe and Asia-an overview. Ticks Tick Borne Dis..

[3] Růžek D, Dobler G, Donoso Mantke O (2010). Tick-borne encephalitis: pathogenesis and clinical implications. Travel Med Infect Dis.

[4] Kunze U (2016). The international scientific working group on tick-borne encephalitis (ISW TBE): review of 17 years of activity and commitment. Ticks Tick Borne Dis.

[5] Robertson SJ, Mitzel DN, Taylor RT, Best SM, Bloom ME (2009). Tick-borne flaviviruses: dissecting host immune responses and virus countermeasures. Immunol Res.

[6] Weststrate AC, Knapen D, Laverman GD, Schot B, Prick JJ, Spit SA, Reimerink J, Rockx B, Geeraedts F (2017). Increasing evidence of tick-borne encephalitis (TBE) virus transmission, the Netherlands, June 2016. Euro Surveill.

[7] Dörrbecker B, Dobler G, Spiegel M, Hufert FT (2010). Tick-borne encephalitis virus and the immune response of the mammalian host. Travel Med Infect Dis.

[8] Brinton MA, Perelygin AA (2003). Genetic resistance to flaviviruses. Adv Virus Res.

[9] Vannberg FO, Chapman SJ, Hill AV (2011). Human genetic susceptibility to intracellular pathogens. Immunol Rev.

[10] Loeb M (2013). Genetic susceptibility to West Nile virus and dengue. Public Health Genomics.

[11] Eslam M, George J (2015). Genome-wide association studies and hepatitis C: harvesting the benefits of the genomic revolution. Semin Liver Dis.

[12] Barkhash AV, Perelygin AA, Babenko VN, Myasnikova NG, Pilipenko PI, Romaschenko AG, Voevoda MI, Brinton MA (2010). Variability in the 2′-5′-oligoadenylate synthetase gene cluster is associated with human predisposition to tick-borne encephalitis virus-induced disease. J Infect Dis.

[13] Barkhash AV, Perelygin AA, Babenko VN, Brinton MA, Voevoda MI (2012). Single nucleotide polymorphism in the promoter region of the *CD209* gene is associated with human predisposition to severe forms of tick-borne encephalitis. Antivir Res.

[14] Barkhash AV, Voevoda MI, Romaschenko AG (2013). Association of single nucleotide polymorphism rs3775291 in the coding region of the *TLR3* gene with predisposition to tick-borne encephalitis in a Russian population. Antivir Res.

[15] Barkhash AV, Babenko VN, Voevoda MI, Romaschenko AG (2016). Association of *IL28B* and *IL10* gene polymorphism with predisposition to tick-borne encephalitis in a Russian population. Ticks Tick-Borne Dis.

[16] Kindberg E, Mickiene A, Ax C, Akerlind B, Vene S, Lindquist L, Lundkvist A, Svensson L (2008). A deletion in the chemokine receptor 5 (CCR5) gene is associated with tickborne encephalitis. J Infect Dis.

[17] Kindberg E, Vene S, Mickiene A, Lundkvist A, Lindquist L, Svensson L (2011). A functional toll-like receptor 3 gene (TLR3) may be a risk factor for tick-borne encephalitis virus (TBEV) infection. J Infect Dis.

[18] Mickienė A, Pakalnienė J, Nordgren J, Carlsson B, Hagbom M, Svensson L, Lindquist L (2014). Polymorphisms in chemokine receptor 5 and toll-like receptor 3 genes are risk factors for clinical tick-borne encephalitis in the Lithuanian population. PLoS One.

[19] Turtle L, Griffiths MJ, Solomon T (2012). Encephalitis caused by flaviviruses. QJM.

[20] Blake LE, Garcia-Blanco MA (2014). Human genetic variation and yellow fever mortality during 19th century U.S. epidemics. MBio.

[21] Bogovic P, Strle F (2015). Tick-borne encephalitis: a review of epidemiology, clinical characteristics, and management. World J Clin Cases.

[22] Yudin NS, Barkhash AV, Maksimov VN, Ignatieva EV, Romaschenko AG. Human genetic predisposition to diseases caused by viruses from Flaviviridae family. Mol Biol (Mosk). 2018;52(2) (in press). 10.7868/S0026898418020039 29695688

[23] Gritsun TS, Lashkevich VA, Gould EA (2003). Tick-borne encephalitis. Antivir Res.

[24] Gritsun TS, Nuttall PA, Gould EA (2003). Tick-borne flaviviruses. Adv Virus Res.

[25] Mandl CW (2005). Steps of the tick-borne encephalitis virus replication cycle that affect neuropathogenesis. Virus Res.

[26] Best SM (2017). The many faces of the Flavivirus NS5 protein in antagonism of type I interferon signaling. J Virol.

[27] Krishnan MN, Ng A, Sukumaran B, Gilfoy FD, Uchil PD, Sultana H, Brass AL, Adametz R, Tsui M, Qian F, Montgomery RR, Lev S, Mason PW, Koski RA, Elledge SJ, Xavier RJ, Agaisse H, Fikrig E (2008). RNA interference screen for human genes associated with West Nile virus infection. Nature.

[28] Mlera L, Lam J, Offerdahl DK, Martens C, Sturdevant D, Turner CV, Porcella SF, Bloom ME (2016). Transcriptome analysis reveals a signature profile for tick-borne Flavivirus persistence in HEK 293T cells. MBio.

[29] Khor CC, Chau TN, Pang J, Davila S, Long HT, Ong RT, Dunstan SJ, Wills B, Farrar J, Van Tram T, Gan TT, Binh NT, Tri le T, Lien le B, Tuan NM, Tham NT, Lanh MN, Nguyet NM, Hieu NT, Van N Vinh Chau N, Thuy TT, Tan DE, Sakuntabhai A, Teo YY, Hibberd ML, Simmons CP (2011). Genome-wide association study identifies susceptibility loci for dengue shock syndrome at MICB and PLCE1. Nat Genet.

[30] Le Breton M, Meyniel-Schicklin L, Deloire A, Coutard B, Canard B, de Lamballerie X, Andre P, Rabourdin-Combe C, Lotteau V, Davoust N (2011). Flavivirus NS3 and NS5 proteins interaction network: a high-throughput yeast two-hybrid screen. BMC Microbiol.

[31] Neal JW (2014). Flaviviruses are neurotropic, but how do they invade the CNS?. J Inf Secur.

[32] Ramage H, Cherry S (2015). Virus-host interactions: from unbiased genetic screens to function. Annu Rev Virol.

[33] Haller O, Kochs G, Weber F (2006). The interferon response circuit: induction and suppression by pathogenic viruses. Virology.

[34] Randall RE, Goodbourn S (2008). Interferons and viruses: an interplay between induction, signalling, antiviral responses and virus countermeasures. J Gen Virol..

[35] Wang BX, Fish EN (2012). The yin and yang of viruses and interferons. Trends Immunol.

[36] Szklarczyk D, Morris JH, Cook H, Kuhn M, Wyder S, Simonovic M, Santos A, Doncheva NT, Roth A, Bork P, Jensen LJ, von Mering C (2017). The STRING database in 2017: quality-controlled protein-protein association networks, made broadly accessible. Nucleic Acids Res.

[37] Smoot ME, Ono K, Ruscheinski J, Wang PL, Ideker T (2011). Cytoscape 2.8: new features for data integration and network visualization. Bioinformatics.

[38] Protopopova EV, Konavalova SN, Loktev VB (1997). Isolation of a cellular receptor for tick-borne encephalitis virus using anti-idiotypic antibodies. Vopr Virusol.

[39] Albornoz A, Carletti T, Corazza G, Marcello A (2014). The stress granule component TIA-1 binds tick-borne encephalitis virus RNA and is recruited to perinuclear sites of viral replication to inhibit viral translation. J Virol.

[40] Dillmann C, Ringel C, Ringleb J, Mora J, Olesch C, Fink AF, Roberts E, Brüne B, Weigert A (2016). S1PR4 signaling attenuates ILT 7 internalization to limit IFN-α production by human Plasmacytoid Dendritic cells. J Immunol.

[41] Ignatieva EV, Levitsky VG, Yudin NS, Moshkin MP, Kolchanov NA (2014). Genetic basis of olfactory cognition: extremely high level of DNA sequence polymorphism in promoter regions of the human olfactory receptor genes revealed using the 1000 genomes project dataset. Front Psychol.

[42] Ignatieva EV, Levitsky VG, Kolchanov NA (2015). Human genes encoding transcription factors and chromatin-modifying proteins have low levels of promoter polymorphism: a study of 1000 genomes project data. Int J Genomics.

[43] Ignatieva EV, Podkolodnaya OA, Orlov YL, Vasiliev GV, Kolchanov NA (2015). Regulatory genomics: integrated experimental and computer approaches. Genetika.

[44] Levitsky VG, Oshchepkov DY, Klimova NV, Ignatieva EV, Vasiliev GV, Merkulov VM, Merkulova TI (2016). Hidden heterogeneity of transcription factor binding sites: a case study of SF-1. Comput Biol Chem.

[45] Aitnazarov RB, Ignatieva EV, Bazarova NE, Levitsky VG, Knyazev SP, Gon Y, Yudin NS (2016). Estimation of the role of single nucleotide polymorphism in Lymphotoxin Beta gene during pig domestication based on the Bioinformatic and experimental approaches. Rus J Genet Appl Res.

[46] Grygorczuk S, Parczewski M, Moniuszko A, Świerzbińska R, Kondrusik M, Zajkowska J, Czupryna P, Dunaj J, Boroń-Kaczmarska A, Pancewicz S (2015). Increased concentration of interferon lambda-3, interferon beta and interleukin-10 in the cerebrospinal fluid of patients with tick-borne encephalitis. Cytokine.

[47] Wigerius M, Melik W, Elväng A, Johansson M (2010). Rac1 and scribble are targets for the arrest of neurite outgrowth by TBE virus NS5. Mol Cell Neurosci.

[48] Upadhyay AS, Vonderstein K, Pichlmair A, Stehling O, Bennett KL, Dobler G, Guo JT, Superti-Furga G, Lill R, Överby AK, Weber F (2014). Viperin is an iron-sulfur protein that inhibits genome synthesis of tick-borne encephalitis virus via radical SAM domain activity. Cell Microbiol.

[49] Yu C, Achazi K, Niedrig M (2013). Tick-borne encephalitis virus triggers inositol-requiring enzyme 1 (IRE1) and transcription factor 6 (ATF6) pathways of unfolded protein response. Virus Res.

[50] Lubick KJ, Robertson SJ, McNally KL, Freedman BA, Rasmussen AL, Taylor RT, Walts AD, Tsuruda S, Sakai M, Ishizuka M, Boer EF, Foster EC, Chiramel AI, Addison CB, Green R, Kastner DL, Katze MG, Holland SM, Forlino A, Freeman AF, Boehm M, Yoshii K, Best SM (2015). Flavivirus antagonism of type I interferon signaling reveals Prolidase as a regulator of IFNAR1 surface expression. Cell Host Microbe.

[51] Kurhade C, Zegenhagen L, Weber E, Nair S, Michaelsen-Preusse K, Spanier J, Gekara NO, Kröger A, Överby AK (2016). Type I interferon response in olfactory bulb, the site of tick-borne flavivirus accumulation, is primarily regulated by IPS-1. J Neuroinflammation.

[52] Tun MM, Aoki K, Senba M, Buerano CC, Shirai K, Suzuki R, Morita K, Hayasaka D (2014). Protective role of TNF-α, IL-10 and IL-2 in mice infected with the Oshima strain of tick-borne encephalitis virus. Sci Rep.

[53] Růžek D, Salát J, Singh SK, Kopecký J (2011). Breakdown of the blood–brain barrier during tick-borne encephalitis in mice is not dependent on CD8+ T-cells. PLoS One.

[54] Barkhash AV, Yurchenko AA, Yudin NS, Ignatieva EV, Kozlova IV, Borishchuk IA, Pozdnyakova LL, Voevoda MI, Romaschenko AG. Matrix metalloproteinase 9 (MMP9) gene single nucleotide polymorphism is associated with predisposition to tick-borne encephalitis virus-induced severe central nervous system disease. Tick Tick Borne Dis. 2018;9. In press. 10.1016/j.ttbdis.2018.02.010 29496490

[55] Carletti T, Zakaria MK, Marcello A (2017). The host cell response to tick-borne encephalitis virus. Biochem Biophys Res Commun.

[56] Guirimand T, Delmotte S, Navratil V (2015). VirHostNet 2.0: surfing on the web of virus/host molecular interactions data. Nucleic Acids Res.

[57] Taylor KE, Mossman KL (2013). Recent advances in understanding viral evasion of type I interferon. Immunology.

[58] Lindqvist R, Mundt F, Gilthorpe JD, Wölfel S, Gekara NO, Kröger A, Överby AK (2016). Fast type I interferon response protects astrocytes from flavivirus infection and virus-induced cytopathic effects. J Neuroinflammation.

[59] Majoros A, Platanitis E, Kernbauer-Hölzl E, Rosebrock F, Müller M, Decker T (2017). Canonical and non-canonical aspects of JAK-STAT signaling: lessons from Interferons for cytokine responses. Front Immunol.

[60] Blanpain C, Migeotte I, Lee B, Vakili J, Doranz BJ, Govaerts C, Vassart G, Doms RW, Parmentier M (1999). CCR5 binds multiple CC-chemokines: MCP-3 acts as a natural antagonist. Blood.

[61] Xiao X, Kinter A, Broder CC, Dimitrov DS (2000). Interactions of CCR5 and CXCR4 with CD4 and gp120 in human blood monocyte-derived dendritic cells. Exp Mol Pathol.

[62] Palus M, Bílý T, Elsterová J, Langhansová H, Salát J, Vancová M, Růžek D (2014). Infection and injury of human astrocytes by tick-borne encephalitis virus. J Gen Virol..

[63] Palus M, Vojtíšková J, Salát J, Kopecký J, Grubhoffer L, Lipoldová M, Demant P, Růžek D (2013). Mice with different susceptibility to tick-borne encephalitis virus infection show selective neutralizing antibody response and inflammatory reaction in the central nervous system. J Neuroinflammation.

[64] Tigabu B, Juelich T, Holbrook MR (2010). Comparative analysis of immune responses to Russian spring-summer encephalitis and Omsk hemorrhagic fever viruses in mouse models. Virology.

[65] Glass WG, Lim JK, Cholera R, Pletnev AG, Gao JL, Murphy PM (2005). Chemokine receptor CCR5 promotes leukocyte trafficking to the brain and survival in West Nile virus infection. J Exp Med.

[66] Regner M, Lobigs M (2012). The chemokine receptor CCR5, a therapeutic target for HIV/AIDS antagonists, is critical for recovery in a mouse model of Japanese encephalitis. PLoS One.

[67] Merzeniuk ZA, Churliaev IA, Nikiforova NV, Kuksinskiĭ VA, Lykova OF, Konysheva TV (2000). The possible role of alpha 2-macroglobulin in regulating the immune components of the brain in tick-borne encephalitis. Zh Mikrobiol Epidemiol Immunobiol.

[68] Havugimana PC, Hart GT, Nepusz T, Yang H, Turinsky AL, Li Z, Wang PI, Boutz DR, Fong V, Phanse S, Babu M, Craig SA, Hu P, Wan C, Vlasblom J, Dar VU, Bezginov A, Clark GW, Wu GC, Wodak SJ, Tillier ER, Paccanaro A, Marcotte EM, Emili A (2012). A census of human soluble protein complexes. Cell.

[69] Yan Z, Cui K, Murray DM, Ling C, Xue Y, Gerstein A, Parsons R, Zhao K, Wang W (2005). PBAF chromatin-remodeling complex requires a novel specificity subunit, BAF200, to regulate expression of selective interferon-responsive genes. Genes Dev.

[70] Lehrer AT, Holbrook MR (2011). Tick-borne encephalitis vaccines. J Bioterror Biodef.

[71] Tonteri E, Kipar A, Voutilainen L, Vene S, Vaheri A, Vapalahti O, Lundkvist Å (2013). The three subtypes of tick-borne encephalitis virus induce encephalitis in a natural host, the bank vole (Myodes glareolus). PLoS One.

[72] Pogodina VV, Bochkova NG, Karan’ LS, Frolova MP, Trukhina AG, Malenko GV, Levina LS, Platonov AE (2004). Comparative analysis of virulence of the Siberian and far-east subtypes of the tick-born encephalitis virus. Vopr Virusol.

[73] Gäumann R, Růžek D, Mühlemann K, Strasser M, Beuret CM (2011). Phylogenetic and virulence analysis of tick-borne encephalitis virus field isolates from Switzerland. J Med Virol.

[74] Asghar N, Lee YP, Nilsson E, Lindqvist R, Melik W, Kröger A, Överby AK, Johansson M (2016). The role of the poly(a) tract in the replication and virulence of tick-borne encephalitis virus. Sci Rep.

[75] Belikov SI, Kondratov IG, Potapova UV, Leonova GN (2014). The relationship between the structure of the tick-borne encephalitis virus strains and their pathogenic properties. PLoS One.

[76] Sakai M, Yoshii K, Sunden Y, Yokozawa K, Hirano M, Kariwa H (2014). Variable region of the 3’ UTR is a critical virulence factor in the far-eastern subtype of tick-borne encephalitis virus in a mouse model. J Gen Virol.

[77] Sakai M, Muto M, Hirano M, Kariwa H, Yoshii K (2015). Virulence of tick-borne encephalitis virus is associated with intact conformational viral RNA structures in the variable region of the 3’-UTR. Virus Res.

[78] Leonova GN, Belikov SI, Kondratov IG, Takashima I (2013). Comprehensive assessment of the genetics and virulence of tick-borne encephalitis virus strains isolated from patients with inapparent and clinical forms of the infection in the Russian far east. Virology.

[79] Luat le X, Tun MM, Buerano CC, Aoki K, Morita K, Hayasaka D (2014). Pathologic potential of variant clones of the oshima strain of far-eastern subtype tick-borne encephalitis virus. Trop Med Health.

[80] Krylova NV, Smolina TP, Leonova GN (2015). Molecular mechanisms of interaction between human immune cells and far eastern tick-borne encephalitis virus strains. Immunol.

[81] Motuzova OV, Akhmatova EA, Khomenkov VG, Akhmatova NK, Lebedinskaya OV, Karganova GG.. Experimental infection caused by two variants of one tick-borne encephalitis virus strain: similar virulence, but different influence on lymphocyte subpopulation structure. Front. Immunol. 2015. Conference Abstract: IMMUNOCOLOMBIA2015 - 11th Congress of the Latin American Association of Immunology - 10o. Congreso de la Asociación Colombiana de Alergia, Asma e Inmunología. doi: 10.3389/conf.fimmu.2015.05.00184.

[82] Cooke GS, Hill AV (2001). Genetics of susceptibility to human infectious disease. Nat Rev Genet..

[83] Hill AV (2006). Aspects of genetic susceptibility to human infectious diseases. Annu Rev Genet.

[84] Newport MJ, Finan C (2011). Genome-wide association studies and susceptibility to infectious diseases. Brief Funct Genomics.

[85] Chapman SJ, Hill AV (2012). Human genetic susceptibility to infectious disease. Nat Rev Genet.

[86] Ellencrona K, Syed A, Johansson M (2009). Flavivirus NS5 associates with host-cell proteins zonula occludens-1 (ZO-1) and regulating synaptic membrane exocytosis-2 (RIMS2) via an internal PDZ binding mechanism. Biol Chem.

[87] Zajkowska JM, Izycka A, Jabłońska E, Hermanowska-Szpakowicz T, Kondrusik M, Pancewicz S, Grygorczuk S, Swierzbińska R (2005). Serum and cerebrospinal concentrations of sICAM-1 sICAM-2, sICAM-3 in neuroborrellosis and tick borne encephalitis - preliminary report. Pol Merkur Lekarski.

[88] Palus M, Zampachová E, Elsterová J, Růžek D (2014). Serum matrix metalloproteinase-9 and tissue inhibitor of metalloproteinase-1 levels in patients with tick-borne encephalitis. J Inf Secur.

[89] Pietruczuk M, Pietruczuk A, Pancewicz S, Zajkowska J, Swierzbińska R, Hermanowska-Szpakowicz T (2006). Intercellular adhesion molecules sICAM-1, sICAM-2, sICAM-3 and IFNgamma in neuroborreliosis and tick-borne encephalitis. Przegl Epidemiol.

[90] Zajkowska J, Moniuszko-Malinowska A, Pancewicz SA, Muszyńska-Mazur A, Kondrusik M, Grygorczuk S, Swierzbińska-Pijanowska R, Dunaj J, Czupryna P (2011). Evaluation of CXCL10, CXCL11, CXCL12 and CXCL13 chemokines in serum and cerebrospinal fluid in patients with tick borne encephalitis (TBE). Adv Med Sci.

[91] Blom K, Braun M, Pakalniene J, Lunemann S, Enqvist M, Dailidyte L, Schaffer M, Lindquist L, Mickiene A, Michaëlsson J, Ljunggren HG, Gredmark-Russ S (2016). NK cell responses to human tick-borne encephalitis virus infection. J Immunol.

[92] Merzeniuk ZA, Lykova OF, Konysheva TV (2003). Lactoferrin and its role in the pathogenesis of tick-borne encephalitis. Klin Lab Diagn.

[93] Kułakowska A, Zajkowska JM, Ciccarelli NJ, Mroczko B, Drozdowski W, Bucki R (2011). Depletion of plasma gelsolin in patients with tick-borne encephalitis and Lyme neuroborreliosis. Neurodegener Dis.

[94] Moniuszko A, Pancewicz S, Czupryna P, Grygorczuk S, Świerzbińska R, Kondrusik M, Penza P, Zajkowska J (2012). ssICAM-1, IL-21 and IL-23 in patients with tick borne encephalitis and neuroborreliosis. Cytokine.

[95] Blom K, Braun M, Pakalniene J, Dailidyte L, Béziat V, Lampen MH, Klingström J, Lagerqvist N, Kjerstadius T, Michaëlsson J, Lindquist L, Ljunggren HG, Sandberg JK, Mickiene A, Gredmark-Russ S (2015). Specificity and dynamics of effector and memory CD8 T cell responses in human tick-borne encephalitis virus infection. PLoS Pathog.

[96] Gomez I, Marx F, Saurwein-Teissl M, Gould EA, Grubeck-Loebenstein B (2003). Characterization of tick-borne encephalitis virus-specific human T lymphocyte responses by stimulation with structural TBEV proteins expressed in a recombinant baculovirus. Viral Immunol.

[97] Aberle JH, Schwaiger J, Aberle SW, Stiasny K, Scheinost O, Kundi M, Chmelik V, Heinz FX (2015). Human CD4+ T helper cell responses after tick-borne encephalitis vaccination and infection. PLoS One.

